# Identifying the effects of education on the ability to cope with a disability among individuals with disabilities

**DOI:** 10.1371/journal.pone.0173659

**Published:** 2017-03-29

**Authors:** Steen Bengtsson, Nabanita Datta Gupta

**Affiliations:** 1 SFI-The Danish National Centre for Social Research, Copenhagen, Denmark; 2 Department of Economics and Business Economics, Aarhus University, Aarhus, Denmark; Universita degli Studi di Perugia, ITALY

## Abstract

The literature on disability has suggested that an educated individual with a disability is more likely to better cope with her/his disability than those without education. However, few published studies explore whether the relationship between education and ability to cope with a disability is anything more than an association. Using data on disability and accommodation from a large Danish survey from 2012–13 and exploiting a major Danish schooling reform as a natural experiment, we identified a potential causal effect of education on both economic (holding a job) as well as social (cultural activities, visiting clubs/associations, etc.) dimensions of coping among individuals with a disability, controlling for background factors, functioning, and disability characteristics. We found that endogeneity bias was only present in the case of economic participation and more educated individuals with a disability indeed had higher levels of both economic and social coping. To some extent, having more knowledge of public support systems and higher motivation explained the better coping among the group of individuals with disabilities who were educated. Our results indicated, however, that a large part of the effect of education on the ability to cope with a disability among individuals with disabilities was suggestive of a causal relationship.

## Introduction

A socioeconomic gradient exists both in disability onset as well as in disability accommodation. A few studies also show that educated individuals with a disability cope better with their disability, i.e. they retain functioning in spite of their impairment and are more likely to be observed working in the labour market. The effect, however, is merely an association. Thus, we do not know whether giving people additional education leads them to cope better with disability, or if education is just a proxy for unobserved traits that correlate positively with coping. For policy purposes it will be important to know whether spillover effects of education exist for disability coping for the purpose of assisting those with partial work incapacity to begin or return to employment [1].

Randomized controlled trials are not always feasible in the social sciences. In the context of the present study, the ideal experiment would be to randomly assign one group of individuals to education early in life but not to another group and then follow both groups over time to see which group copes better with a disability later in life. Since this is not ethically advisable (i.e. denying one group a fundamental human right) and in addition would require a very long observation period, we exploit instead what is known as a “quasi-natural experiment” in this paper [2]. Natural experiments and quasi-natural experiments mimic random assignment, for example, if one group of individuals is subject to either a natural calamity, accident, unanticipated reform or other sharp change in their circumstance which is outside their control (the treated) while an observationally equivalent group has not experienced it (the controls). Any difference in outcomes between the two groups can thus be plausibly attributed to the natural or social experiment. This method of identification is widely used in the social sciences for making (potential) causal inference [3] [4].

Using this approach, we are one of the first to provide reliable evidence on whether education (potentially) causally affects disability coping using a large Danish randomized survey on disabled and abled from 2012–13. The Danish setting is an interesting one because the universal health care system gives individuals with a disability free access to most primary physician services and acute care, free public support, and tax-financed education. This implies that, if a socioeconomic gradient in disability accommodation is found, it is much less likely to be due to differential access to health care, welfare support or education than in other countries. However, even though all education (including tertiary education) in Denmark is tuition-free and tax-financed, children from lower socioeconomic backgrounds may be less likely to participate in higher education due to lower levels of cultural capital growing up and a lack of role models [5]. At the same time, Danish workplaces are among the most accommodating in the world, with municipalities covering necessary adaptations, wage subsidy programs for employers or disabled workers, and opportunities for sick-listed workers for partial return to work without risk of benefit loss [6]. Thus, the effects of socioeconomic background on disability coping found in a Danish setting most likely represent a lower bound of what could be expected in other settings.

Our focus will be on identifying a potential causal effect of education on disability coping, and on uncovering the precise channels through which educated individuals with a disability better cope with their disability. For example, the educated could be more efficient at combining inputs or they could have greater health knowledge. One U.S. study shows that among elderly disabled the better educated are less affected by their impairment. The study further finds that elderly disabled who are educated tend to use more assistive technologies and more paid help. However, these factors do not explain why they can better cope with their disability [7].

In this study, we use representative Danish survey data sampling the working-age population. After estimating our models using quasi-experimental econometric methodology exploiting a major school reform that exogenously increased the level of education only for certain groups, we consider several pathways or mechanisms through which any potential causal effects arise. Instead of examining the use of assistive technology at home which the previous literature found played only a limited role, we consider whether educated persons with a disability may more easily be granted *workplace adaptations*. We add to the literature by empirically examining whether educated individuals with a disability may have an *informational advantage* in terms of working the system (i.e. knowing how and where to obtain free counseling, access to handicap organizations, benefits, etc.). Finally, we also control for the potential effect of educated individuals with a disability individuals having higher levels of *intrinsic motivation*. The rich survey data at our disposal has information on the degree to which individuals with a disability use each of these resources.

Disability is illuminated in several alternative ways in our study–having restricted the sample to those with a self-reported long-term physical or mental disability, we control in the analyses for the type (mental or not), self reported severity, duration (proportion of one’s life with a disability), inborn or not, as well as functioning captured via ADLs and IADLs. Note, individuals in our sample with a mental disability reporting having a slight cognitive impairment and not a severe intellectual disability or a learning disability. ADLs (Activities of Daily Living) and IADLs (Instrumental Activities of Daily Living) are standard measures of functioning and capture how well the individual manages the basic activities of daily living such as eating and dressing and a more expanded set of activities such as shopping, housework, financial and administrative tasks etc. Although the measure for selecting individuals to participate in the sample is based on a self-report of a long-standing condition, the detailed nature of the questions in the sample (i.e., first probing the existence of a disability, then querying individuals with a disability about many dimensions of the disability) reduces any measurement error. Furthermore, self-reported health has been shown to be an excellent predictor of objective health, for instance, global self-rated health is an important and independent predictor of mortality found consistently across 27 different studies from the US and other settings [8]. We investigate not only whether educated individuals with a disability have a higher probability of being employed, but also whether they are better off according to an alternative measure of well-being, i.e. participating in either social, cultural, nightlife, meetings in associations, volunteer activities, or night school on at least a monthly basis. Gannon and Nolan [9] find that the onset of a disability is associated with a decline in the probability of employment and, especially in the case of a severe disability, also hampers social participation.

The previous literature finds ample evidence of an education gradient in (the presence of) disability. Crimmins and Saito [10] examine healthy life expectancy and thereby indirectly examine acquired disability in the U.S. in 1970, 1980, and 1990. They find that these educational differences have been increasing over time. Schoeni et al. [11] also find that the socioeconomic gradient in disability for the elderly grew in the 1982–2002 period. Melzer et al. [12] find that elderly men with up to seven years of education have a risk of disability 65% higher than men with 12 or more years of education, and the corresponding risk for women is 70% higher. They find no difference, however, between educational groups in the chances of recovery or in the risk of death once disabled. Economic means are important for acquiring and coping with a disability, see e.g. Zimmer and House [13]. In the Scandinavian setting, however, the public sector freely provides the needed assistive technology, paid help, and home modifications. Thus, other mechanisms are likely to be more relevant for the educational gradient. We add to this literature by examining whether an educational gradient exists for disability coping, whether the effect is potentially causal, and what the likely mechanisms behind such an effect are.

In terms of a theoretical basis, the theory of social inclusion is a fundamental concept in sociology literature on inequality. Social inclusion is seen as essential for living a meaningful life, including having self-reliance and motivation to live and stay healthy. Social inclusion takes the form of social contacts, leisure activities, participation in employment, and participation in other informal social contexts. Gannon & Nolan [9], analyzing movement in and out of disability as well as persistent disability, have found that disability entails a much lower degree of social inclusion. Despite the fact that a large literature has built up around coping with health conditions in the medical, nursing and public health areas, the notion of “coping” in general is broad and not well defined; in this case, it refers to the actions undertaken to manage the stressful situations caused by chronic illness or disability [14]. Various social models of coping behaviour have been proposed, ranging from individualist coping (personal agency theories, factors such as individual age, gender, and personality) to social support as a means of coping [15]. Another view proposes a conservation of resources approach, such that compensating actions will be undertaken to try to minimize the effects of any loss [16]. In line with these three theories, we propose three mechanisms that may potentially give rise to causal effect of education on coping–an individual’s intrinsic motivation which proxies personal agency, (knowledge of) social support systems that measures the use of social support, and workplace accommodative practices capturing compensating actions. Furthermore, we include age, gender, cohabitation status, health endowment (i.e. parental mortality) and current health (disability characteristics, ADLs and IADLs) as control variables in the analysis.

In health economics literature, Grossman’s [17] theory of health conceives of health as a durable capital stock, in which individuals combine market inputs and time inputs to invest optimally in restoring health deterioration. Grossman also derives the demand for medical care from the more fundamental demand for “good health.” Grossman’s theory can be used to explain some of the social inequalities in health. Considering health stock and health-promoting differences, and equating marginal benefit to marginal cost, it explains why educated individuals, in general, are likely to be healthier than uneducated individuals. Within the framework of this model as well, obtaining and using knowledge of support systems and seeking out accommodation at the workplace are ways in which educated people would invest more in their health, because their expected returns to healthy days exceed those of uneducated people. Whereas, intrinsic motivation may capture a selection effect, i.e. that persons who are future-oriented (have a higher time preference for the future rather than the present time) acquire both more education and invest more in their health [18].

A few studies employing robust identification methods have uncovered a potentially causal negative relationship between education and disability, meaning that education does indeed reduce disability and is not simply a proxy for unobserved traits. (See [19] and [20], who use self-reported disability; and [21], who use a benefits-receipt measure.) Examining the antecedents of disability coping sheds light on that literature as well.

## Methods

Data on disability and its accommodation are taken from a 2012–13 Danish Web and telephone survey known as the SHILD (Survey of Health, Impairment and Living Conditions in Denmark). These data consist of a random sample of around 33,000 persons aged 16 to 64 conducted by The Danish National Institute of Social Research (SFI) during October 2012–February 2013 [22]. A total of 32,810 persons between the ages 16 and 64 were randomly drawn from the Central Person Register. Out of these persons there were 18,957 respondents, constituting 57.8% of the gross sample. The number of persons with the highest degree of functional limitation tallies with national medical estimates for every type of disability except for mental disability, for which the high degrees of functional limitation are under-represented in the survey (most of this particular group did not respond to the survey).

In terms of validation, 25.1% of individuals in the survey data report having a disability. (The question read: “Do you suffer from a long-term physical health problem or disability?”). In comparison, according to the Swedish Labor Force surveys, 19–21% report a disability in Sweden [23], while a Norwegian study reports that 18% have an identifiable impairment [24]. The first OPCS survey of disability from Britain from 1988 found that 13.5% of the population had a long-standing and limiting disability [25]. The more recent EU SILC finds in 2013 that 19% of the 18–64 population in the EU and 26.3% in Denmark has self-reported activity limitation [26]. The latter number confirms our result.

After identifying individuals with a disability, the survey contained an additional question about the degree of disability and the extent of public support for this group. Survey data carry a great advantage over administrative data in this context. Disability is much more widespread and is associated with a greater reduction in work capacity than the more serious but rarely occurring health events recorded in register data. The survey records not just the presence of a disability but the severity as well, meaning that even minor impairments are included but will not carry much weight.

The respondents answered a number of questions in a broad range of topics. In this analysis, we use data on education, gender, age, cohabitation, parents’ mortality, disability characteristics and functioning, motivation, knowledge of the system (i.e. disability councils, public services), and adaptation at the workplace. These data have been merged and supplemented with 15 years of register data on accurately measured education and other labor-market characteristics, meaning that work status is known with accuracy.

## Results

We measure education as registered school education greater than nine years. A key school reform of the educational system from 1975 (affecting the cohorts born in 1962 or later) dramatically changed opportunities for participating in more than nine years of education by abolishing preparatory upper middle school (*realskole*) and allowing regular schools to offer 8^th^ to 9^th^ grades.

Before this reform, schools determined on the basis of an exam after 7^th^ grade whether students would attend the two-year high school-preparatory upper middle school track or an ordinary school-leaving track. The reform significantly eased access to high school for students in regular schools.

We exploit this reform to generate an exogenous increase in the probability of obtaining more than compulsory school level education (>9 years) for the affected cohorts. (Overall 84% of the sample have more than 9 years of education, see Table A in S1 Appendix. This figure is 83% for the pre-reform cohorts and 87% for the post-reform cohorts). Thus, we instrument education by being born in the reform cohorts (1962 or later)–this is the variable labelled ‘Affected by the reform.’ We restrict the sample to only include the 10 cohorts born before and after the reform (1952–1971). Of our sample, 43% are in the age-group who is affected by the reform. However, to account for a general rising enrollment in education over time, we also control for month of birth (‘mob’) in the instrument regression, and furthermore ‘Affected by the reform*mob’ to allow for a differential cohort trend before and after the reform.

**Fig 1:**
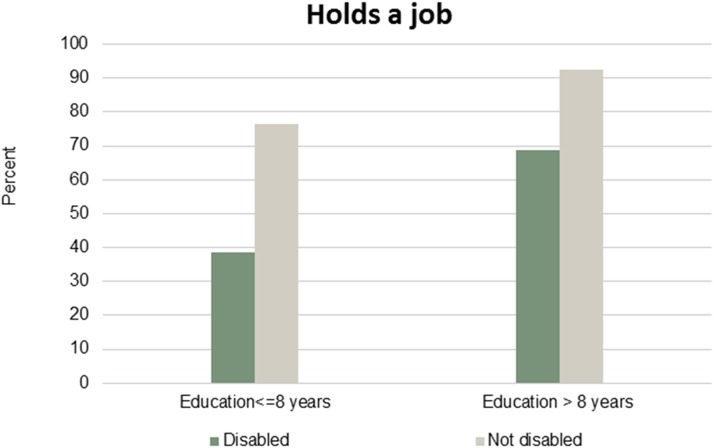
Holds a job. Percent who holds a job by education and disability.

**Fig 2:**
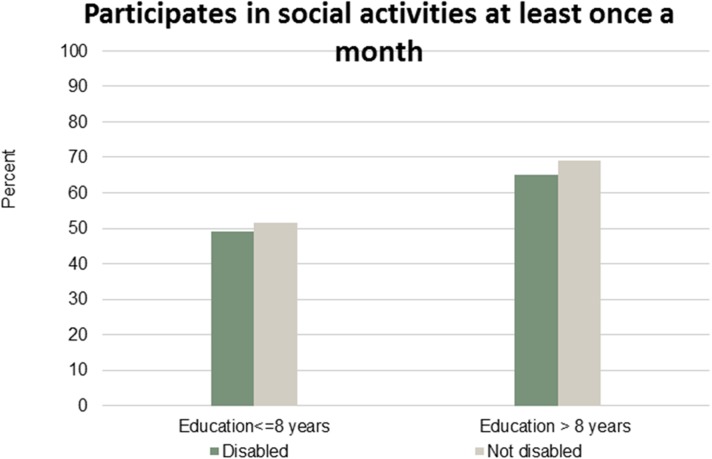
Participates in social activities at least once a month. Percent who participates in social activities at least once a month by education and disability.

**Fig 3:**
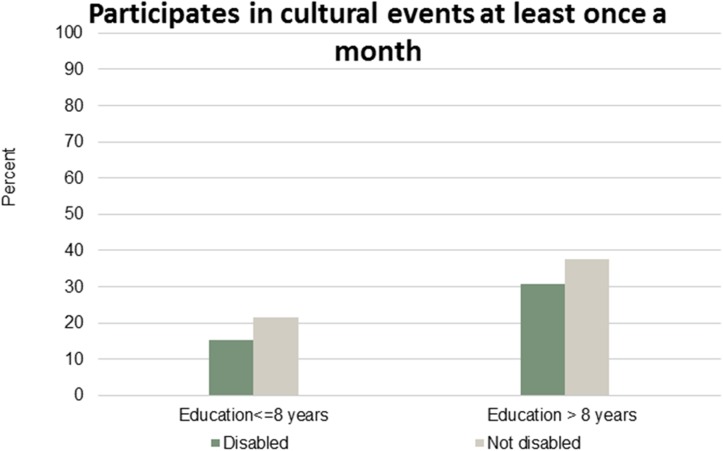
Participates in cultural events at least once a month. Percent who participates in cultural events at least once a month by education and disability.

**Fig 4:**
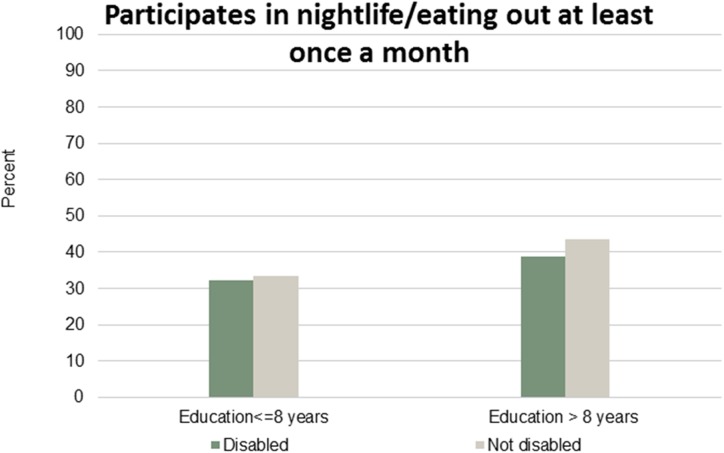
Participates in night life/eating out at least once a month. Percent who participates in night life/eating out at least once a month by education and disability.

**Fig 5:**
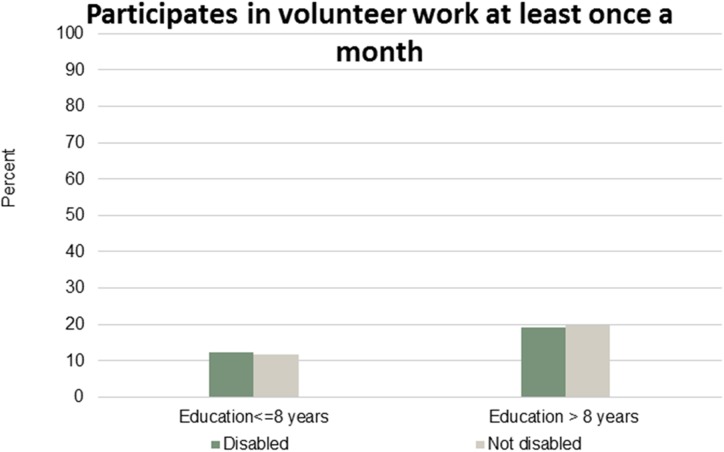
Participates in volunteer work at least once a month. Percent who participates in volunteer work at least once a month by education and disability.

**Fig 6:**
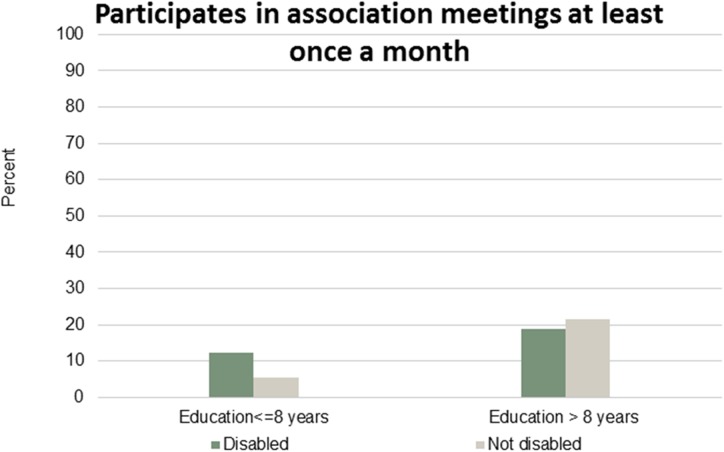
Participates in association meetings at least once a month. Percent who participates in association meetings at least once a month by education and disability.

**Fig 7:**
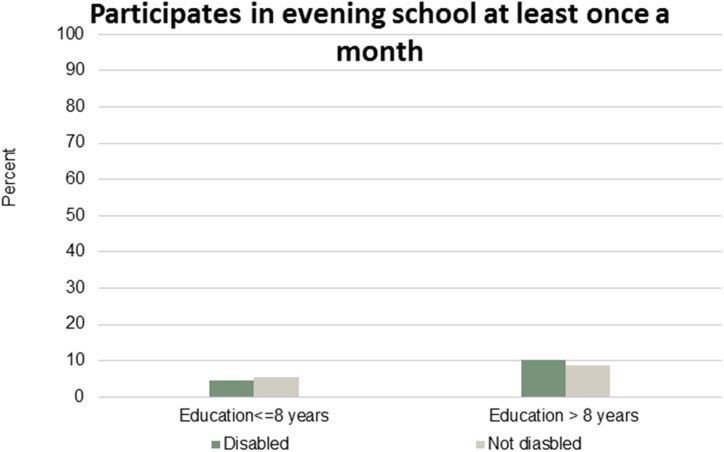
Participates in evening school at least once a month. Percent who participates in evening school at least once a month by education and disability.

Figs 1–7 show that the relationship between education and the employment rate is positive, and a steeper educational gradient is seen among individuals with a disability compared to abled. From the next set of figures, Figs 2–7 we do not see any clear educational gradient regarding any dimension of social participation except for participating in cultural events. In almost all cases it can be seen that individuals with a disability are less socially active than individuals without a disability.

Table A in S1 Appendix contains the basic descriptives of the sample of 2,814 disabled persons on which we based the empirical analysis. Of individuals with a disability, 68% of the sample holds a job while 65% have at least some form of monthly social participation. The most popular activity is eating out/nightlife, which 39% experience. Of the sample, 84% have at least nine years of basic education, 58% are female, 75% live with a partner, 58% have a living mother, while the share with a living father is much lower at 38%. The mean age in the sample is 51 and ranges from 40 to 60 by construction. Recall, we limited ourselves to only 10 birth cohorts before and after the reform of 1975. The most frequently reported functional difficulty is walking up 12 steps and the most frequent instrumental difficulty is doing housework and taking public transportation (about 8% each report having this difficulty). The disability severity score is on average 0.35 (ranges from 0 to 1), and on average individuals have had their disability for a little more than a quarter of their lives. Of the sample, 23% report a mental disability whereas only 2% are born with their disability; 38% have knowledge of counselling agencies, 43% have knowledge of public support services, and 28% access adaptive arrangements at the workplace. Of the respondents, 13% have low levels of motivation, and finally 43% of the individuals in our sample are affected by the 1975 reform.

In Tables B-C in S1 Appendix, we test with a series of regressions whether the positive association between education and the various aspects of social participation is suggestive of a causal relationship. We describe first the control variables in the models. The *background factors* are age, sex, cohabitation/marriage, and whether the individual’s mother and/or father are alive. *Health and functioning* comprise ADLs and IADLs, type of disability (mental or not), the severity score of the disability, the proportion of one’s life one has had the disability, and whether it is an inborn disability or not. *Knowledge of the social support system* comprises two indices, one of them summing up knowledge of counseling agencies and the other summing up knowledge of different public support schemes. Note that more system knowledge might itself reflect improved functioning (e.g. obtaining the necessary information by going on the computer or going around to offices). The variable *motivation* comprises a single question asking a disabled individual whether he or she perceives his or her daily existence as being empty and meaningless. The survey included four related questions on motivational state, but as their correlation was high, we retained only the one correlating most with the three others.

We estimate four models for each of our two outcomes, employment and social participation. In Model 1 only background factors and the month of birth trend (‘mob’) are included in the X vector. In Model 2 background factors, ‘mob’, plus health and functioning are included in the X vector. Model 3 contains the same controls as in Model 2 except that educational attainment is simultaneously modelled with employment using a bivariate probit model, exploiting belonging in the reform cohorts as an instrument and controlling for both a jump at the threshold, a common trend and different trends on either side of the threshold. Model 4 is the same as Model 3 except that knowledge of couselling agencies, knowledge of public support services and motivation are included in addition in the main outcome equation. This is done to explore possible mechanisms behind any potential causal effect. Since our dependent variables are 0/1 variables, we estimate probit models in the first two columns and bivariate probit models in the next two columns.

The probit model is a regression model for binary dependent variables in which the error term is assumed to take on the standard normal distribution. The results are shown in terms of marginal effects (population-averaged) and can be interpreted as showing how much an additional unit of a continuous right-hand side variable (or how much going from 0 to 1 for a right hand side dummy variable) increases the probability of coping with a disability measured in percentage points. When modelling educational attainment jointly with disability coping the bivariate probit model is used, which enables simultaneous estimation of two interdependent binary variables. That is, while in the univariate case, education is treated as exogenously given, in the bivariate case we estimate the decision to study more than 9 years jointly with the coping equation. We choose to take into account the binary nature of the dependent variables and therefore we estimate probit and biprobit models rather than linear IV models. The bivariate probit model can be estimated in the presence of an endogeneous variable. Although a unique instrument is not technically required in the bivariate probit, it aids in identification. In this case, the instrument is the reform variable.

One may be concerned that many outcomes (including employment, education, and social participation) are improving over time for individuals with disabilities; and that cohorts who attended school after 1975 would do better on these outcomes than those who attended school previously regardless of the educational reform. To take this into account, we have added the variable mob (month of birth, which takes unique values for each month and year of birth) in the regression, plus mob interacted with being in the reform cohorts. Thus, these variables allow for a smooth trend in the outcome variables plus a differential trend on either side of the reform threshold.

Starting first with the results in Table B in S1 Appendix, we see that when controlling for background factors, having only at least basic education raises the probability of the disabled holding a job by 22 percentage points and is highly significant (Column 1). However, this effect becomes smaller although still significant at the 5% level when health and functioning are included, the effect size being halved to 13 percentage points. Thus, greater work participation is partly due to better health and functioning. In Column 3 where the endogeneity of education is taken into account, we see that the instrument is positive and significant and that the effect of education is now stronger, 45 percentage points, and still significant. The estimate of ‘rho’ at the bottom of Column 3 indicates that endogeneity cannot be ruled out at the 5% level.

Taking the endogeneity into account makes the effect of education on job holding stronger indicating that individuals with a disability with more education are negatively selected in terms of their unobserved characteristics. This can be seen in the estimate of ‘rho,’ which is negative indicating that the unobservables in employment vary negatively with the unobservables in educational attainment. Finally, in the last column where we consider possible mechanisms, we see a 5–15% reduction in the effect of education on employment mainly due to including motivation, although knowledge of especially public support services also appears to exert a significant effect and increases the employment rate by nearly 2 percentage points. The effect of motivation is strong and highly significant. Having low motivation reduces the employment rate by 7.3 percentage points.

We conclude that education has a potential causal effect on the employment rate among the disabled. The effect size in Column 4 is relatively large, around 50% of the baseline employment rate. Furthermore, we conclude that some part of this effect could be arising because educated people are in general more motivated. Whether this represents individuals’ intrinsic motivation levels which made them acquire an education in the first place (selection) or whether acquiring an education has made these individuals more motivated cannot be resolved within this framework. However, since motivation is operationalized as a desire to remain in the workforce, it may plausibly represent the latter.

The second group of regressions in Table C in S1 Appendix considers participation relating to the social sphere, i.e. the dependent variable is 1 if there is participation in any one of the dimensions described earlier, such as cultural activities, eating out/nightlife, volunteer work, visiting clubs and associations or going to night school at least once a month. The models do not explain much, and the education effect is less than for holding a job, but still significant in the first two models. Having at least a basic education has a positive marginal effect of 13–16 percentage points on the likelihood of any form of social participation (20–25% of baseline). Other than education, for social participation we see that disability characteristics, motivation, and knowledge of the system are important for explaining the education gradient.

However, when modelling educational attainment simultaneously, the effect becomes insignificant, although it remains positive. The instrument is also less significant here and the estimate of ‘rho’ is significant only at the 10% level. Thus, there is not a strong case for endogeneity in this case, which is why we prefer the estimates of Model 2. In terms of mechanisms, we see controlling for knowledge of the system (public support schemes), and low motivation reduces the effect of education in Model 2 slightly by 1.9 percentage points, meaning that educated persons possess more of this factor.

In Table D in S1 Appendix we disaggregate social participation in its separate dimensions and run the same models as above. Only in the case of cultural participation do we find an education effect. The effect is similar to what we found in Table C in S1 Appendix when looking overall at social participation. Thus, education mainly affects participation in cultural activities among individuals with a disability and the effect is more than 50% of the baseline rate. For the other dimensions, education has a positive though insignificant effect.

In terms of the control variables, a few factors that stand out in these models is living with a partner (affects employment only positively), having a living mother (affects social participation only positively), and several of the IADLs and ADLs and having a mental disability (affects both outcomes negatively). Finally, in Table E in S1 Appendix as a robustness test we omit individuals with an inborn disability from the sample. This is to avoid having the disability affecting the amount of educational investments undertaken starting early in life and onwards. We can see from this exercise that our results for both economic and social participation remain robust to this exclusion.

## Conclusion

Previous research shows that education reduces the risk of acquiring a disability and that this correlation expresses a potential causal relation. Earlier observational research also finds that educated individuals with a disability seem to cope or function better with their disability. Thus far, no studies have been able to establish whether there exists a potentially causal effect between education and disability coping, and furthermore, what the concrete mechanisms are behind this relationship.

In this paper, we considered two forms of coping while being disabled, i.e. being employed (economic participation) and participating in various dimensions of social life (social participation). Controlling for age, gender, cohabitation, and parental longevity, we find that education has a positive association with both such measures of participation. Where holding a job is concerned, we find evidence of endogeneity bias in the estimated effect of education. After accounting for endogeneity, there is a potential causal effect of education, which is only partly affected by having high motivation and knowledge of the public support systems, but not by knowledge of counselling agencies or workplace adaptations. When considering social participation we find little evidence of endogeneity, and the single equation model results show that the effect of education is again affected but in a limited way by knowledge of the public support system and motivation, but not by knowledge of counseling agencies or workplace adaptations. The main aspect of social participation that is affected by education is participation in cultural activities.

Our aim was to shed light on the relationship between education and disability and find the concrete mechanisms underlying it. We conclude that education has an effect on formal participation in the labour market and social participation especially in cultural arrangements. The potential causal relationship between education and participation arises partly due to knowledge of the system and motivation, but not by workplace adaptation. Thus this evidence seems to align more with social support and individualistic coping mechanisms suggested by the theory rather than the compensatory actions mechanism. However, the effect does not seem to be influenced much by the inclusion of the former two variables, implying that it is mainly the effect of the extra education obtained as a result of the reform that affects disability coping later in life. Whether this is due to the actual educational content of the studies or to education changing individuals’ preferences is a matter we leave for future research.

To return to the unanswered question in earlier literature of how the better educated are better able to cope with their disability, we can conclude that this mainly represents an effect of education itself and not of either more knowledge of the system, higher levels of intrinsic motivation, or a better work environment among educated people. However, we should point out certain limitations of our study, i.e., that the disability measure is self-reported, the analysis method only quasi-experimental and the sample limited to individuals with a disability. Nonetheless, the share who reports themselves as being disabled in our survey closely matches the share disabled in Denmark in the 2013 EU-SILC survey. Furthermore, one advantage with using a self-reported measure is that it allows us to also capture disabilities that do not require hospitalization, as opposed to a disability measure created on the basis of medical reports. Finally, to the extent that our findings are suggestive of a causal effect, the implication for policy would be to continue to strive to increase the educational attainment of the population. In Denmark, around 20% of youths do not complete more than the 9 years of compulsory education. The results of this paper show that this group would experience difficulties coping with a disability later in life.

## Supporting information

S1 Appendix Appendix A. Variable definitions and tables A-E.
(DOCX)
