# Supplementary material for: Identifying the effects of education on the ability to cope with a disability among individuals with disabilities
Source: PLoS One. 2017 Mar 29;12(3):e0173659. doi: 10.1371/journal.pone.0173659 (PMC5371281; doi:10.1371/journal.pone.0173659)
Supplement: S1 Appendix — Variable definitions and tables A-E. (DOCX) [file pone.0173659.s001.docx]

**Appendix: Variable definitions and tables A-E**

**Variable Definitions**

| Outcomes: |  |
| --- | --- |
| Holds a job | 1= holds an ordinary or a supported job, 0 else |
| Social participation | 1= social contacts outside the household at least once a week, 0 else |
| Social participation: Cultural events | 1= goes to cultural or sports events at least once a month, 0 else |
| Social participation: Nightlife/eating out | 1= goes out in the city at least once a month, 0 else |
| Social participation: Volunteer work | 1= does volunteer work at least once a month, 0 else |
| Social participation: Meetings in associations | 1= goes to meetings in associations at least once a month, 0 else |
| Social participation: Evening school | 1= uses evening school at least once a month, 0 else |
| Controls: |  |
| Education: > 9 years | 1= completed at least 9 years of compulsory school |
| Gender (Female=1) | 1= female, 0= male |
| Living with partner | 1= living with partner or married, 0 else |
| Mother alive | 1= mother alive, 0 else |
| Father alive | 1= father alive, 0 else |
| Age | Age in years |
| Month of birth | Continuous measure of cohort based on year and month of birth, ranging from 0 to 239 |
| Difficulty with: Seeing | 1= self-reported difficulty with seeing, 0 else |
| Difficulty with: Hearing | 1= self-reported difficulty with hearing, 0 else |
| Difficulty with: Walking 100 meters | 1= self-reported difficulty with walking, 0 else |
| Difficulty with: Walking up 12 steps | 1= self-reported difficulty with steps, 0 else |
| Difficulty with: Concentration | 1= self-reported difficulty with concentration, 0 else |
| Difficulty with: Eating | 1= self-reported difficulty with eating, 0 else |
| Difficulty with: Dressing | 1= self-reported difficulty with dressing, 0 else |
| Difficulty with: Shopping | 1= self-reported difficulty with shopping, 0 else |
| Difficulty with: Housework | 1= self-reported difficulty with housework, 0 else |
| Difficulty with: Using public transport | 1= self-reported difficulty with taking bus and train, 0 else |
| Difficulty with: Financial and administrative tasks | 1= self-reported difficulty with financial and administrative tasks, 0 else |
| Difficulty with: Structuring everyday life | 1= self-reported difficulty with structuring everyday life, 0 else |
| Difficulty with: Reading or spelling | 1= self-reported difficulty with reading and spelling, 0 else |
| Disability severity score | 1= self-reported serious disability, 0 else |
| Proportion of life have had disability | (age – age aquiring disability) / age |
| Mental disability | 1= has mental disability, 0 else |
| Inborn disability | 1= disability was inborn, 0 else |
| Knowledge of counselling agencies | 1= has knowledge of counselling agencies, 0 else |
| Knowledge of public support services | 1= has knowledge of public support services, 0 else |
| Adaption at workplace | 1= has been granted adaptations at workplace, 0 else |
| Low motivation | 1= has a wish of withdrawing from work, 0 else |
| Affected by reform in 1975 | 1= belongs to a cohort born 1952-1971, 0 else |

**Table A. Descriptive Statistics**

| **Outcome variables** |  |  |  |  |  |
| --- | --- | --- | --- | --- | --- |
|  |  |  |  |  |  |
|  | **Observations** | **Mean** | **Std. deviation** | **Min** | **Max** |
| Holds a job | 2814 | 0.6866 | 0.4640 | 0 | 1 |
| Social participation | 2813 | 0.6495 | 0.4772 | 0 | 1 |
| Social participation: Cultural events | 2814 | 0.3063 | 0.4610 | 0 | 1 |
| Social participation: Nightlife/eating out | 2814 | 0.3866 | 0.4871 | 0 | 1 |
| Social participation: Volunteer work | 2813 | 0.1913 | 0.3934 | 0 | 1 |
| Social participation: Meetings in associations | 2814 | 0.1876 | 0.3905 | 0 | 1 |
| Social participation: Evening school | 2814 | 0.1031 | 0.3041 | 0 | 1 |
| **Control variables** |  |  |  |  |  |
|  |  |  |  |  |  |
|  | **Observations** | **Mean** | **Std. deviation** | **Min** | **Max** |
| Education: > 9 years | 2814 | 0.8444 | 0.3626 | 0 | 1 |
| Gender (Female=1) | 2814 | 0.5780 | 0.4937 | 0 | 1 |
| Living with partner | 2814 | 0.7484 | 0.4340 | 0 | 1 |
| Mother alive | 2814 | 0.5796 | 0.4937 | 0 | 1 |
| Father alive | 2814 | 0.3724 | 0.4835 | 0 | 1 |
| Age | 2814 | 51.18 | 5.6370 | 40 | 60 |
| Month of birth | 2814 | 107.5 | 67.59 | 0 | 239 |
| Difficulty with: Seeing | 2814 | 0.02416 | 0.1536 | 0 | 1 |
| Difficulty with: Hearing | 2814 | 0.01990 | 0.1397 | 0 | 1 |
| Difficulty with: Walking 100 meters | 2814 | 0.04549 | 0.2084 | 0 | 1 |
| Difficulty with: Walking up 12 steps | 2814 | 0.06219 | 0.2415 | 0 | 1 |
| Difficulty with: Concentration | 2814 | 0.04691 | 0.2115 | 0 | 1 |
| Difficulty with: Eating | 2814 | 0.001422 | 0.03768 | 0 | 1 |
| Difficulty with: Dressing | 2814 | 0.01031 | 0.1010 | 0 | 1 |
| Difficulty with: Shopping | 2814 | 0.03554 | 0.1852 | 0 | 1 |
| Difficulty with: Housework | 2814 | 0.08138 | 0.2735 | 0 | 1 |
| Difficulty with: Using public transport | 2814 | 0.08884 | 0.2846 | 0 | 1 |
| Difficulty with: Financial and administrative tasks | 2814 | 0.03909 | 0.1938 | 0 | 1 |
| Difficulty with: Structuring everyday life | 2814 | 0.01955 | 0.1385 | 0 | 1 |
| Difficulty with: Reading or spelling | 2814 | 0.01706 | 0.1296 | 0 | 1 |
| Disability severity score | 2814 | 0.3472 | 0.4762 | 0 | 1 |
| Proportion of life have had disability | 2814 | 0.2632 | 0.2478 | 0 | 1 |
| Mental disability | 2814 | 0.2328 | 0.4227 | 0 | 1 |
| Inborn disability | 2814 | 0.01706 | 0.1295 | 0 | 1 |
| Knowledge of counselling agencies | 2814 | 0.3838 | 0.4864 | 0 | 1 |
| Knowledge of public support services | 2814 | 0.4282 | 0.4949 | 0 | 1 |
| Adaption at workplace | 2814 | 0.2758 | 0.4470 | 0 | 1 |
| Low motivation | 2814 | 0.1329 | 0.3395 | 0 | 1 |
| Affected by reform in 1975 | 2814 | 0.4289 | 0.4950 | 0 | 1 |

**Table B. Probit and Biprobit Regressions of Economic Participation**

| **Dependent variable: Holds a job** |  |  |  |  |  |  |
| --- | --- | --- | --- | --- | --- | --- |
|  |  |  |  |  |  |  |
|  | Probit | | Biprobit | | | |
|  | **Model 1** | **Model 2** | **Model 3** | *Instrument reg* | **Model 4** | *Instrument reg* |
| Education: > 9 years | 0.216*** | 0.129** | 0.413*** | *Dependent var* | 0.359** | *Dependent var* |
|  | (0.0559) | (0.0487) | (0.107) |  | (0.120) |  |
| Gender (Female=1) | -0.0853*** | -0.0615*** | -0.0620*** | *0.00575* | -0.0644*** | *0.00647* |
|  | (0.0171) | (0.0151) | (0.0149) | *(0.00549)* | (0.0151) | *(0.00553)* |
| Living with partner | 0.175*** | 0.103*** | 0.100*** | *0.000527* | 0.0936*** | *0.000464* |
|  | (0.0180) | (0.0163) | (0.0161) | *(0.00619)* | (0.0163) | *(0.00618)* |
| Mother alive | 0.0292 | 0.0218 | 0.0194 | *0.00445* | 0.0165 | *0.00421* |
|  | (0.0184) | (0.0160) | (0.0158) | *(0.00602)* | (0.0159) | *(0.00602)* |
| Father alive | 0.0236 | 0.0144 | 0.0127 | *0.00805* | 0.0133 | *0.00787* |
|  | (0.0198) | (0.0173) | (0.0171) | *(0.00765)* | (0.0171) | *(0.00765)* |
| Month of birth | 0.000625*** | 0.000704*** | 0.000631*** | *0.000690**** | 0.000683*** | *0.000677**** |
|  | (0.000143) | (0.000127) | (0.000127) | *(0.000133)* | (0.000130) | *(0.000132)* |
| Difficulty with: Seeing |  | 0.0138 | 0.0222 | *-0.0207* | 0.0224 | *-0.0232* |
|  |  | (0.0511) | (0.0502) | *(0.0127)* | (0.0506) | *(0.0126)* |
| Difficulty with: Hearing |  | -0.00880 | -0.0163 | *0.226* | -0.0118 | *0.212* |
|  |  | (0.0528) | (0.0522) | *(136.9)* | (0.0521) | *(63.71)* |
| Difficulty with: Walking 100 meters |  | -0.157** | -0.146** | *-0.0129* | -0.142** | *-0.0145* |
|  |  | (0.0542) | (0.0534) | *(0.0143)* | (0.0534) | *(0.0142)* |
| Difficulty with: Walking up 12 steps |  | -0.0404 | -0.0297 | *-0.0260** | -0.0288 | *-0.0271** |
|  |  | (0.0423) | (0.0419) | *(0.0124)* | (0.0422) | *(0.0123)* |
| Difficulty with: Concentration |  | -0.0724 | -0.0739 | *0.0139* | -0.0625 | *0.0147* |
|  |  | (0.0399) | (0.0393) | *(0.0157)* | (0.0394) | *(0.0157)* |
| Difficulty with: Eating |  | 0.381* | 0.347* | *0.238* | 0.353* | *0.243* |
|  |  | (0.177) | (0.176) | *(112.4)* | (0.178) | *(69.57)* |
| Difficulty with: Dressing |  | 0.0171 | 0.0101 | *0.00158* | 0.00523 | *-0.00538* |
|  |  | (0.103) | (0.101) | *(0.0244)* | (0.102) | *(0.0230)* |
| Difficulty with: Shopping |  | -0.0761 | -0.0754 | *0.00171* | -0.0756 | *-0.000490* |
|  |  | (0.0555) | (0.0543) | *(0.0154)* | (0.0546) | *(0.0151)* |
| Difficulty with: Housework |  | -0.158*** | -0.155*** | *0.00407* | -0.147*** | *0.000250* |
|  |  | (0.0329) | (0.0324) | *(0.0128)* | (0.0326) | *(0.0127)* |
| Difficulty with: Using public transport |  | -0.134*** | -0.135*** | *0.0271** | -0.132*** | *0.0259* |
|  |  | (0.0315) | (0.0310) | *(0.0136)* | (0.0310) | *(0.0134)* |
| Difficulty with: Financial and administrative tasks |  | -0.131** | -0.130** | *-0.00577* | -0.119* | *-0.00400* |
|  |  | (0.0488) | (0.0479) | *(0.0159)* | (0.0481) | *(0.0165)* |
| Difficulty with: Structuring everyday life |  | -0.165* | -0.156* | *-0.0182* | -0.146 | *-0.0203* |
|  |  | (0.0810) | (0.0791) | *(0.0193)* | (0.0792) | *(0.0197)* |
| Difficulty with: Reading or spelling |  | -0.177** | -0.153* | *-0.0549**** | -0.163* | *-0.0514**** |
|  |  | (0.0684) | (0.0671) | *(0.0150)* | (0.0675) | *(0.0147)* |
| Severe disability |  | -0.189*** | -0.184*** | *-0.00471* | -0.179*** | *-0.00474* |
|  |  | (0.0147) | (0.0147) | *(0.00613)* | (0.0153) | *(0.00622)* |
| Proportion of life with disability |  | -0.0611 | -0.0620 | *-0.000195* | -0.0658* | *0.00173* |
|  |  | (0.0327) | (0.0322) | *(0.0128)* | (0.0323) | *(0.0128)* |
| Mental disability |  | -0.120*** | -0.117*** | *-0.000529* | -0.112*** | *-0.00269* |
|  |  | (0.0171) | (0.0169) | *(0.00673)* | (0.0171) | *(0.00672)* |
| Inborn disability |  | 0.0875 | 0.0882 | *0.00471* | 0.0836 | *0.00391* |
|  |  | (0.0677) | (0.0665) | *(0.0258)* | (0.0662) | *(0.0259)* |
| Knowledge of disability councils |  |  |  |  | 0.000470 | *0.00456* |
|  |  |  |  |  | (0.0159) | *(0.00580)* |
| Knowledge of public services |  |  |  |  | 0.0172 | *0.0105* |
|  |  |  |  |  | (0.0156) | *(0.00600)* |
| Low motivation |  |  |  |  | -0.0730*** | *0.0248** |
|  |  |  |  |  | (0.0216) | *(0.0113)* |
| Adaption at workplace |  |  |  |  | 0.00791 | *-0.00764* |
|  |  |  |  |  | (0.0164) | *(0.00588)* |
| Affected by reform in 1975 |  |  |  | *14.09*** |  | *13.69*** |
|  |  |  |  | *(5.084)* |  | *(5.068)* |
| Affected by reform in 1975* mob |  |  |  | *-0.000600*** |  | *-0.000583*** |
|  |  |  |  | *(0.000216)* |  | *(0.000215)* |
|  |  |  |  |  |  |  |
| N | 2814 | 2814 | 2814 | | 2814 | |
| Log-likelihood | -1662.2 | -1321.8 | -1569.3 | | -1556.9 | |
| Chi2 (ll) | 175.2*** | 856.0*** | 759.2*** | | 755.2*** | |
| MacFadden’s Pseudo R^2^ | 0.050 | 0.245 |  | |  | |
| Rho |  |  | -0.514 | | -0.420 | |
| p-value (likelihood test of rho=0) |  |  | 0.0274 | | 0.1006 | |

Education is instrumented by reform, mob og reform*mob in biprobit models

Coefficients are AME (average marginal effects), standard errors in parentheses, * p<0.05, ** p<0.01, *** p<0.001

**Table C. Probit and Biprobit Regressions of Social Participation**

| **Dependent variable: Social participation** | | |  |  |  |  |  |
| --- | --- | --- | --- | --- | --- | --- | --- |
|  |  |  |  |  |  |  |  |
|  | Probit | | | Biprobit | | | |
|  | **Model 1** | **Model 2** | **Model 3** | **Model 4** | *Instrument reg* | **Model 5** | *Instrument reg* |
| Education: > 9 years | 0.164** | 0.134* | 0.128* | 0.325 | *Dependent var* | 0.170 | *Dependent var* |
|  | (0.0593) | (0.0586) | (0.0582) | (0.340) |  | (0.309) |  |
| Gender (Female=1) | 0.0135 | 0.0197 | 0.0171 | 0.0183 | *0.00631* | 0.0168 | *0.00745* |
|  | (0.0182) | (0.0180) | (0.0182) | (0.0181) | *(0.00545)* | (0.0184) | *(0.00549)* |
| Living with partner | 0.0117 | -0.0127 | -0.0215 | -0.0132 | *0.00118* | -0.0216 | *0.000787* |
|  | (0.0207) | (0.0208) | (0.0209) | (0.0207) | *(0.00635)* | (0.0209) | *(0.00644)* |
| Mother alive | 0.0474* | 0.0432* | 0.0395* | 0.0413* | *0.00431* | 0.0391* | *0.00411* |
|  | (0.0197) | (0.0194) | (0.0194) | (0.0196) | *(0.00599)* | (0.0196) | *(0.00600)* |
| Father alive | -0.0200 | -0.0229 | -0.0235 | -0.0236 | *0.00900* | -0.0236 | *0.00865* |
|  | (0.0209) | (0.0205) | (0.0205) | (0.0204) | *(0.00760)* | (0.0205) | *(0.00766)* |
| Month of birth | -0.0000934 | -0.0000452 | 0.0000320 | -0.0000873 | *0.000641**** | 0.0000215 | *0.000638**** |
|  | (0.000154) | (0.000154) | (0.000155) | (0.000167) | *(0.000128)* | (0.000172) | *(0.000127)* |
| Difficulty with: Seeing |  | 0.0404 | 0.0353 | 0.0456 | *-0.0174* | 0.0366 | *-0.0217* |
|  |  | (0.0589) | (0.0587) | (0.0590) | *(0.0134)* | (0.0594) | *(0.0134)* |
| Difficulty with: Hearing |  | 0.134* | 0.134* | 0.127 | *0.205* | 0.133 | *0.196* |
|  |  | (0.0684) | (0.0675) | (0.0689) | *(38.07)* | (0.0683) | *(17.13)* |
| Difficulty with: Walking 100 meters |  | -0.0773 | -0.0762 | -0.0738 | *-0.0157* | -0.0754 | *-0.0144* |
|  |  | (0.0597) | (0.0596) | (0.0597) | *(0.0144)* | (0.0598) | *(0.0143)* |
| Difficulty with: Walking up 12 steps |  | 0.0335 | 0.0337 | 0.0419 | *-0.0219* | 0.0356 | *-0.0237* |
|  |  | (0.0516) | (0.0513) | (0.0533) | *(0.0127)* | (0.0530) | *(0.0127)* |
| Difficulty with: Concentration |  | -0.0576 | -0.0396 | -0.0594 | *0.0190* | -0.0402 | *0.0173* |
|  |  | (0.0463) | (0.0464) | (0.0460) | *(0.0175)* | (0.0465) | *(0.0165)* |
| Difficulty with: Eating |  | -0.148 | -0.112 | -0.163 | *0.217* | -0.116 | *0.221* |
|  |  | (0.282) | (0.275) | (0.282) | *(166.8)* | (0.276) | *(66.30)* |
| Difficulty with: Dressing |  | -0.0302 | -0.0357 | -0.0347 | *0.00660* | -0.0366 | *-0.00220* |
|  |  | (0.0967) | (0.0973) | (0.0963) | *(0.0242)* | (0.0974) | *(0.0228)* |
| Difficulty with: Shopping |  | -0.192*** | -0.190** | -0.191*** | *0.00520* | -0.190** | *0.000406* |
|  |  | (0.0582) | (0.0583) | (0.0579) | *(0.0154)* | (0.0582) | *(0.0152)* |
| Difficulty with: Housework |  | 0.00234 | 0.00909 | 0.00196 | *0.00137* | 0.00904 | *-0.00142* |
|  |  | (0.0408) | (0.0409) | (0.0405) | *(0.0122)* | (0.0409) | *(0.0121)* |
| Difficulty with: Using public transport |  | -0.0342 | -0.0300 | -0.0376 | *0.0197* | -0.0308 | *0.0193* |
|  |  | (0.0389) | (0.0388) | (0.0390) | *(0.0125)* | (0.0391) | *(0.0124)* |
| Difficulty with: Financial and administrative tasks |  | -0.0121 | -0.00297 | -0.0130 | *-0.00233* | -0.00306 | *-0.000377* |
|  |  | (0.0529) | (0.0529) | (0.0525) | *(0.0155)* | (0.0529) | *(0.0160)* |
| Difficulty with: Structuring everyday life |  | -0.155* | -0.151* | -0.149* | *-0.0225* | -0.150* | *-0.0225* |
|  |  | (0.0743) | (0.0742) | (0.0750) | *(0.0195)* | (0.0747) | *(0.0201)* |
| Difficulty with: Reading or spelling |  | -0.0913 | -0.0966 | -0.0733 | *-0.0516**** | -0.0930 | *-0.0487**** |
|  |  | (0.0713) | (0.0706) | (0.0781) | *(0.0146)* | (0.0754) | *(0.0143)* |
| Severe disability |  | -0.0503* | -0.0412* | -0.0483* | *-0.00558* | -0.0408 | *-0.00611* |
|  |  | (0.0203) | (0.0208) | (0.0205) | *(0.00649)* | (0.0210) | *(0.00630)* |
| Proportion of life with disability |  | -0.00471 | -0.00887 | -0.00516 | *0.00229* | -0.00902 | *0.00512* |
|  |  | (0.0391) | (0.0390) | (0.0388) | *(0.0129)* | (0.0390) | *(0.0129)* |
| Mental disability |  | -0.0657** | -0.0619** | -0.0648** | *0.0000650* | -0.0617** | *-0.00269* |
|  |  | (0.0215) | (0.0216) | (0.0214) | *(0.00691)* | (0.0216) | *(0.00694)* |
| Inborn disability |  | 0.144 | 0.130 | 0.142 | *-0.000224* | 0.130 | *-0.00232* |
|  |  | (0.0786) | (0.0783) | (0.0782) | *(0.0239)* | (0.0782) | *(0.0241)* |
| Knowledge of disability councils |  |  | 0.00720 |  |  | 0.00694 | *0.00469* |
|  |  |  | (0.0192) |  |  | (0.0193) | *(0.00580)* |
| Knowledge of public services |  |  | 0.0663*** |  |  | 0.0657*** | *0.0118* |
|  |  |  | (0.0187) |  |  | (0.0193) | *(0.00616)* |
| Low motivation |  |  | -0.0547* |  |  | -0.0555* | *0.0249** |
|  |  |  | (0.0273) |  |  | (0.0279) | *(0.0113)* |
| Adaption at workplace |  |  | 0.00251 |  |  | 0.00286 | *-0.00874* |
|  |  |  | (0.0204) |  |  | (0.0205) | *(0.00580)* |
| Affected by reform in 1975 |  |  |  |  | *12.57** |  | *12.42** |
|  |  |  |  |  | *(4.903)* |  | *(4.938)* |
| Affected by reform in 1975* mob |  |  |  |  | *-0.000535** |  | *-0.000529** |
|  |  |  |  |  | *(0.000208)* |  | *(0.000210)* |
|  |  |  |  |  |  |  |  |
| N | 2813 | 2813 | 2813 | 2813 | | 2813 | |
| Log-likelihood | -1814.6 | -1766.3 | -1756.7 | -2016.1 | | -1999.5 | |
| Chi2 (ll) | 15.12* | 111.7*** | 130.9*** | 191.7*** | | 210.1*** | |
| MacFadden’s Pseudo R^2^ | 0.004 | 0.031 | 0.036 |  | |  | |
| Rho |  |  |  | -0.252 | | -0.0561 | |
| p-value (likelihood test of rho=0) |  |  |  | 0.5949 | | 0.8905 | |

Education is instrumented by reform, mob og reform*mob in biprobit models

Coefficients are AME (average marginal effects), standard errors in parentheses, * p<0.05, ** p<0.01, *** p<0.001

**Table D. Probit Regressions of Various Spheres of Social Participation**

|  |  |  |  |
| --- | --- | --- | --- |
| **Dependent variable: Participation in cultural events** | | | |
|  |  |  |  |
|  | Probit | | |
|  | Model 1 | Model 2 | Model 3 |
| Education: > 9 years | 0.220** | 0.188** | 0.178* |
|  | (0.0711) | (0.0711) | (0.0709) |
|  |  |  |  |
| N | 2814 | 2814 | 2814 |
| Log-likelihood | -1727.3 | -1677.1 | -1668.7 |
| Chi2 (ll) | 13.06* | 113.5*** | 130.2*** |
| Pseudo R2 | 0.004 | 0.033 | 0.038 |
| Rho |  |  |  |
| p-value (likelihood test of rho=0) |  |  |  |
|  |  |  |  |
| **Dependent variable: Participation in nightlife/eating out** |  |  |  |
|  | Probit | | |
|  | Model 1 | Model 2 | Model 3 |
| Education: > 9 years | 0.0992 | 0.0840 | 0.0835 |
|  | (0.0642) | (0.0641) | (0.0639) |
|  |  |  |  |
| N | 2814 | 2814 | 2814 |
| Log-likelihood | -1869.5 | -1843.8 | -1836.9 |
| Chi2 (ll) | 16.02* | 67.44*** | 81.29*** |
| Pseudo R2 | 0.004 | 0.018 | 0.022 |
| Rho |  |  |  |
| p-value (likelihood test of rho=0) |  |  |  |
|  |  |  |  |
| **Dependent variable: Participation in volunteer work** | | | |
|  |  |  |  |
|  | Probit | | |
|  | Model 1 | Model 2 | Model 3 |
| Education: > 9 years | 0.0979 | 0.0892 | 0.0812 |
|  | (0.0578) | (0.0581) | (0.0580) |
|  |  |  |  |
| N | 2813 | 2813 | 2813 |
| Log-likelihood | -1367.3 | -1349.0 | -1330.3 |
| Chi2 (ll) | 11.02 | 47.79** | 85.19*** |
| Pseudo R2 | 0.004 | 0.017 | 0.031 |
| Rho |  |  |  |
| p-value (likelihood test of rho=0) |  |  |  |
|  |  |  |  |
| **Dependent variable: Participation in association meetings** | | | |
|  |  |  |  |
|  | Probit | | |
|  | Model 1 | Model 2 | Model 3 |
| Education: > 9 years | 0.0747 | 0.0657 | 0.0654 |
|  | (0.0488) | (0.0494) | (0.0492) |
|  |  |  |  |
| N | 2814 | 2814 | 2814 |
| Log-likelihood | -913.0 | -901.2 | -898.1 |
| Chi2 (ll) | 41.06*** | 64.60*** | 70.94*** |
| Pseudo R2 | 0.022 | 0.035 | 0.038 |
| Rho |  |  |  |
| p-value (likelihood test of rho=0) |  |  |  |
|  |  |  |  |
| **Dependent variable: Participation in evening school** |  |  |  |
|  | Probit | | |
|  | Model 1 | Model 2 | Model 3 |
| Education: > 9 years | 0.0702 | 0.0385 | 0.0290 |
|  | (0.0548) | (0.0542) | (0.0536) |
|  |  |  |  |
| N | 2814 | 2814 | 2814 |
| Log-likelihood | -1341.8 | -1318.3 | -1300.0 |
| Chi2 (ll) | 33.36*** | 80.45*** | 116.9*** |
| Pseudo R2 | 0.012 | 0.030 | 0.043 |
| Rho |  |  |  |
| p-value (likelihood test of rho=0) |  |  |  |
| Coefficients are AME (average marginal effects) | | | |
| Standard errors in parentheses | |  |  |
| * p<0.05, ** p<0.01, *** p<0.001 | |  |  |

**Table E. Probit and Biprobit Regressions of Economic and Social Participation, Excluding those with inborn disability**

| **Dependent variable: Holds a job** | | |  |  |  |
| --- | --- | --- | --- | --- | --- |
| (excluding those with an inborn disability) | | | |  |  |
|  | Probit | | | Biprobit | |
|  | **Model 1** | **Model 2** |  | **Model 3** | **Model 4** |
| Education: > 9 years | 0.231*** | 0.151** |  | 0.463*** | 0.414*** |
|  | (0.0565) | (0.0496) |  | (0.103) | (0.116) |
|  |  |  |  |  |  |
| N | 2766 | 2766 |  | 2766 | 2766 |
| Log-likelihood | -1632.6 | -1304.7 |  | -1546.7 | -1534.5 |
| Chi2 (ll) | 173.1*** | 829.1*** |  | 754.1*** | 749.8*** |
| Pseudo R2 | 0.050 | 0.241 |  |  |  |
| Rho |  |  |  | -0.569 | -0.486 |
| p-value (likelihood test of rho=0) |  |  |  | 0.0174 | 0.0639 |
|  |  |  |  |  |  |
| **Dependent variable: Social participation** | | |  |  |  |
| (excluding those with an inborn disability) | | | |  |  |
|  | Probit | | | Biprobit | |
|  | **Model 1** | **Model 2** | **Model 3** | **Model 4** | **Model 5** |
| Education: > 9 years | 0.171** | 0.139* | 0.133* | 0.312 | 0.168 |
|  | (0.0598) | (0.0592) | (0.0588) | (0.344) | (0.306) |
|  |  |  |  |  |  |
| N | 2765 | 2765 | 2765 | 2765 | 2765 |
| Log-likelihood | -1786.2 | -1740.0 | -1730.5 | -1984.7 | -1968.6 |
| Chi2 (ll) | 15.71* | 108.0*** | 127.1*** | 187.7*** | 206.1*** |
| Pseudo R2 | 0.004 | 0.030 | 0.035 |  |  |
| Rho |  |  |  | -0.229 | -0.0465 |
| p-value (likelihood test of rho=0) |  |  |  | 0.6254 | 0.9079 |
| Education is instrumented by reform, mob og reform*mob in biprobit models |  |  |  |  |  |
| Controlling for same variables as in Tables 2 & 3 | | | |  |  |
| Coefficients are AME (average marginal effects) | | | |  |  |
| Standard errors in parentheses | | |  |  |  |
| * p<0.05, ** p<0.01, *** p<0.001 | | |  |  |  |
